# 2-[6-Thioxo-5-(2,4,6-trimethyl­phen­yl)-1,3,5-thia­diazinan-3-yl]acetic acid

**DOI:** 10.1107/S1600536809011027

**Published:** 2009-03-28

**Authors:** Mohammad Arfan, M. Nawaz Tahir, Muhammad Ishaq Ali Shah, Rassol Khan, Mohammad S. Iqbal

**Affiliations:** a Institute of Chemical Sciences, University of Peshawar, Peshawar 25120, Pakistan; b Department of Physics, University of Sargodha, Sargodha, Pakistan; c Department of Chemistry, Government College University, Lahore, Pakistan

## Abstract

In the mol­ecule of the title compound, C_14_H_18_N_2_O_2_S_2_, the 1,3,5-thia­diazinane-2-thione ring adopts an envelope conformation with one of the N atoms at the flap position. The plane throught the five co-planar atoms of the heterocycle is oriented at a dihedral angle of 80.59 (8)° with respect to the aromatic ring. In the crystal structure, weak inter­molecular O—H⋯S inter­actions link the mol­ecules into chains along the *b* axis.

## Related literature

For related structures, see: Arfan *et al.* (2009 ▶); Perez *et al.* (2001 ▶). For bond-length data, see: Allen *et al.* (1987 ▶). 
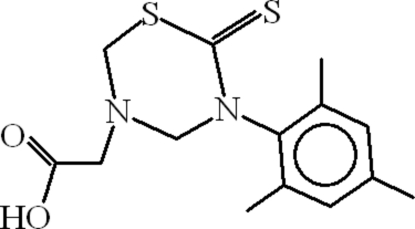


## Experimental

### 

#### Crystal data

C_14_H_18_N_2_O_2_S_2_
*M* *_r_* = 310.42
Orthorhombic, 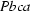

*a* = 6.9134 (4) Å
*b* = 17.6934 (11) Å
*c* = 24.9073 (15) Å
*V* = 3046.7 (3) Å^3^
*Z* = 8
Mo *K*α radiation
μ = 0.35 mm^−1^
*T* = 296 K
0.26 × 0.18 × 0.16 mm

#### Data collection

Bruker Kappa APEXII CCD area-detector diffractometer
Absorption correction: multi-scan (*SADABS*; Bruker, 2005 ▶) *T*_min_ = 0.922, *T*_max_ = 0.942
18229 measured reflections
3957 independent reflections
2120 reflections with *I* > 2σ(*I*)
*R*_int_ = 0.053

#### Refinement

*R*[*F* ^2^ > 2σ(*F* ^2^)] = 0.046
*wR*(*F* ^2^) = 0.132
*S* = 1.03
3957 reflections
186 parameters
H-atom parameters constrained
Δρ_max_ = 0.32 e Å^−3^
Δρ_min_ = −0.27 e Å^−3^

### 

Data collection: *APEX2* (Bruker, 2007 ▶); cell refinement: *SAINT* (Bruker, 2007 ▶); data reduction: *SAINT*; program(s) used to solve structure: *SHELXS97* (Sheldrick, 2008 ▶); program(s) used to refine structure: *SHELXL97* (Sheldrick, 2008 ▶); molecular graphics: *ORTEP-3 for Windows* (Farrugia, 1997 ▶) and *PLATON* (Spek, 2009 ▶); software used to prepare material for publication: *WinGX* (Farrugia, 1999 ▶) and *PLATON*.

## Supplementary Material

Crystal structure: contains datablocks global, I. DOI: 10.1107/S1600536809011027/hk2650sup1.cif

Structure factors: contains datablocks I. DOI: 10.1107/S1600536809011027/hk2650Isup2.hkl

Additional supplementary materials:  crystallographic information; 3D view; checkCIF report

## Figures and Tables

**Table 1:** Hydrogen-bond geometry (Å, °)

*D*—H⋯*A*	*D*—H	H⋯*A*	*D*⋯*A*	*D*—H⋯*A*
O1—H1⋯S2^i^	0.82	2.37	3.187 (2)	174

Symmetry code: (i) 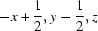.

**Fig. 1.**
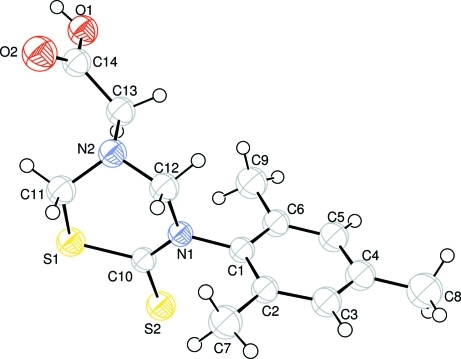
The molecular structure of the title molecule, with the atom-numbering scheme.

**Fig. 2.**
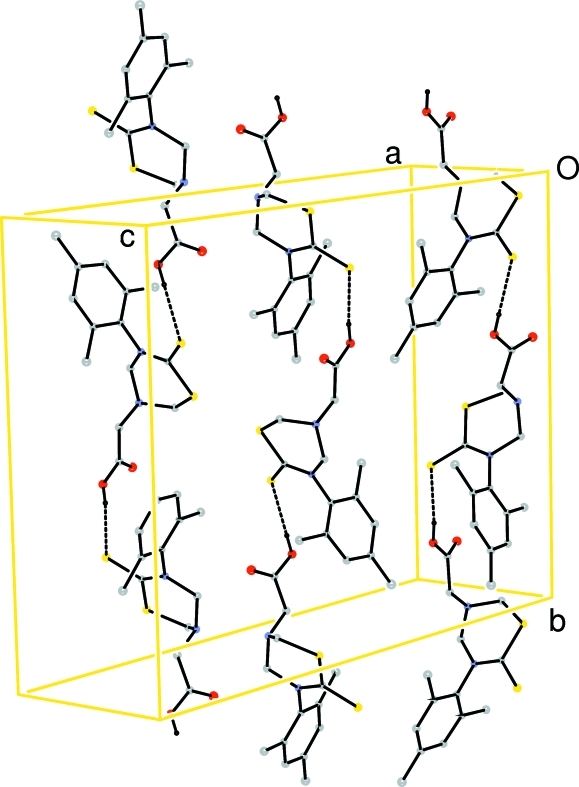
A partial packing diagram of the title compound. Hydrogen bonds are shown as dashed lines. Hydrogen atoms not involved in hydrogen bonding have been omitted.

## supplementary crystallographic information

### Comment

We have recently reported the crystal structure of 3-benzyl-5-butyl-1,3,5 -thiadiazinane-2-thione, (II) (Arfan *et al.*,  2009) and 5-carboxyethyl-3 -(2'-furfurylmethyl) tetrahydro-2*H*-1,3,5-thiadiazine-2-thione, (III) (Perez *et al.*,  2001) has also been published. As part of our ongoing studies, we report herein the crystal structure of the title compound, (I).

In the molecule of the title compound (Fig. 1), the bond lengths (Allen *et al.*,  1987) and angles are within normal ranges. Ring A (C1-C6) is, of course, planar, while ring B (S1/N1/N2/C10-C12) adopts an envelope conformation with atom N2 displaced by -0.647 (3) Å from the plane of the other ring atoms. The planar carboxylic acid moiety is oriented with respect to ring A at a dihedral angle of 34.26 (3)°.

In the crystal structure, weak intermolecular O-H···S interactions (Table 1) link the molecules into chains along the b axis (Fig. 2), in which they may be effective in the stabilization of the structure.

### Experimental

For the preparation of the title compound, carbon disulfide was slowly added into a mixture of 2,4,6-trimethylaniline (2.18 ml, 20 mmol) and potassium hydroxide (20%, 20 mmol) in water (30 ml) with stirring. Formaldehyde (37%) was added dropwise to the mixture after 4 h, and was stirred for a further 1 h. Then, the mixture was filtered and the filtrate was added in a suspension of glycine (1.5 ml, 20 mmol) prepared in phosphate buffer solution (20 ml, pH = 7.8), and stirred for a further 1 h. The reaction mixture was filtered and extracted with dichloromethane. The aqueous solution was acidified using HCl and the precipitates formed were filtered and washed with water. The residues were recrystallized in ethanol by slow evaporation (yield; 75%, m. p. 421-423 K).

### Refinement

H atoms were positioned geometrically, with O-H = 0.82 Å (for OH) and C-H = 0.93, 0.97 and 0.96 Å for aromatic, methylene and methyl H, respectively, and constrained to ride on their parent atoms, with U_iso_(H) = xU_eq_(C,O), where x = 1.5 for methyl H and x = 1.2 for all other H atoms.

### Crystal data

**Table:**

C_14_H_18_N_2_O_2_S_2_	*F*(000) = 1312
*M**_r_* = 310.42	*D*_x_ = 1.354 Mg m^−^^3^
Orthorhombic, *P**b**c**a*	Mo *K*α radiation, λ = 0.71073 Å
Hall symbol: -P 2ac 2ab	Cell parameters from 3957 reflections
*a* = 6.9134 (4) Å	θ = 2.4–28.8°
*b* = 17.6934 (11) Å	µ = 0.35 mm^−^^1^
*c* = 24.9073 (15) Å	*T* = 296 K
*V* = 3046.7 (3) Å^3^	Prism, colorless
*Z* = 8	0.26 × 0.18 × 0.16 mm

### Data collection

**Table:**

Bruker Kappa APEXII CCD area-detector diffractometer	3957 independent reflections
Radiation source: fine-focus sealed tube	2120 reflections with *I* > 2σ(*I*)
graphite	*R*_int_ = 0.053
Detector resolution: 7.40 pixels mm^-1^	θ_max_ = 28.8°, θ_min_ = 2.4°
ω scans	*h* = −9→6
Absorption correction: multi-scan (*SADABS*; Bruker, 2005)	*k* = −24→21
*T*_min_ = 0.922, *T*_max_ = 0.942	*l* = −32→33
18229 measured reflections	

### Refinement

**Table:**

Refinement on *F*^2^	Primary atom site location: structure-invariant direct methods
Least-squares matrix: full	Secondary atom site location: difference Fourier map
*R*[*F*^2^ > 2σ(*F*^2^)] = 0.046	Hydrogen site location: inferred from neighbouring sites
*wR*(*F*^2^) = 0.132	H-atom parameters constrained
*S* = 1.03	*w* = 1/[σ^2^(*F*_o_^2^) + (0.0418*P*)^2^ + 2.0698*P*] where *P* = (*F*_o_^2^ + 2*F*_c_^2^)/3
3957 reflections	(Δ/σ)_max_ = 0.001
186 parameters	Δρ_max_ = 0.32 e Å^−^^3^
0 restraints	Δρ_min_ = −0.27 e Å^−^^3^

### Special details

**Table:**

Geometry. Bond distances, angles *etc*. have been calculated using the rounded fractional coordinates. All su's are estimated from the variances of the (full) variance-covariance matrix. The cell e.s.d.'s are taken into account in the estimation of distances, angles and torsion angles
Refinement. Refinement of *F*^2^ against ALL reflections. The weighted *R*-factor *wR* and goodness of fit *S* are based on *F*^2^, conventional *R*-factors *R* are based on *F*, with *F* set to zero for negative *F*^2^. The threshold expression of *F*^2^ > σ(*F*^2^) is used only for calculating *R*-factors(gt) *etc*. and is not relevant to the choice of reflections for refinement. *R*-factors based on *F*^2^ are statistically about twice as large as those based on *F*, and *R*- factors based on ALL data will be even larger.

### Fractional atomic coordinates and isotropic or equivalent isotropic displacement parameters (Å^2^)

**Table:**

	*x*	*y*	*z*	*U*_iso_*/*U*_eq_	
S1	0.05204 (10)	0.04895 (4)	0.06455 (3)	0.0462 (3)	
S2	−0.05342 (10)	0.17988 (4)	0.12532 (3)	0.0502 (3)	
O1	0.4812 (3)	−0.14565 (11)	0.15003 (8)	0.0521 (7)	
O2	0.5343 (4)	−0.12709 (11)	0.06276 (9)	0.0639 (9)	
N1	0.3157 (3)	0.14751 (11)	0.10199 (8)	0.0342 (7)	
N2	0.4407 (3)	0.02387 (12)	0.07081 (9)	0.0409 (8)	
C1	0.3789 (4)	0.21613 (14)	0.12872 (11)	0.0355 (8)	
C2	0.4250 (4)	0.27902 (14)	0.09806 (11)	0.0382 (9)	
C3	0.4943 (4)	0.34268 (15)	0.12500 (13)	0.0465 (10)	
C4	0.5158 (4)	0.34412 (16)	0.18049 (13)	0.0494 (10)	
C5	0.4705 (4)	0.27966 (16)	0.20908 (13)	0.0508 (11)	
C6	0.4040 (4)	0.21442 (15)	0.18430 (12)	0.0423 (9)	
C7	0.3984 (5)	0.28082 (17)	0.03821 (12)	0.0554 (11)	
C8	0.5854 (5)	0.41477 (18)	0.20860 (15)	0.0693 (14)	
C9	0.3599 (5)	0.14439 (18)	0.21674 (12)	0.0617 (11)	
C10	0.1280 (4)	0.13078 (13)	0.09810 (10)	0.0356 (8)	
C11	0.2787 (4)	0.01152 (15)	0.03629 (12)	0.0455 (10)	
C12	0.4777 (4)	0.10269 (14)	0.07749 (13)	0.0451 (10)	
C13	0.4446 (4)	−0.02219 (15)	0.11916 (11)	0.0465 (10)	
C14	0.4921 (4)	−0.10347 (15)	0.10630 (13)	0.0444 (10)	
H1	0.49414	−0.19027	0.14189	0.0625*	
H3	0.52712	0.38540	0.10524	0.0556*	
H5	0.48496	0.27999	0.24620	0.0610*	
H7A	0.43567	0.32952	0.02473	0.0833*	
H7B	0.47751	0.24253	0.02198	0.0833*	
H7C	0.26508	0.27157	0.02968	0.0833*	
H8A	0.62761	0.45096	0.18238	0.1037*	
H8B	0.48151	0.43591	0.22931	0.1037*	
H8C	0.69113	0.40233	0.23195	0.1037*	
H9A	0.35779	0.15697	0.25423	0.0926*	
H9B	0.23599	0.12472	0.20634	0.0926*	
H9C	0.45771	0.10699	0.21028	0.0926*	
H11A	0.30305	0.03556	0.00195	0.0545*	
H11B	0.26428	−0.04230	0.03002	0.0545*	
H12A	0.59162	0.10850	0.09985	0.0542*	
H12B	0.50755	0.12413	0.04261	0.0542*	
H13A	0.54067	−0.00223	0.14376	0.0558*	
H13B	0.31956	−0.01976	0.13673	0.0558*	

### Atomic displacement parameters (Å^2^)

**Table:**

	*U*^11^	*U*^22^	*U*^33^	*U*^12^	*U*^13^	*U*^23^
S1	0.0368 (4)	0.0420 (4)	0.0599 (5)	−0.0050 (3)	−0.0129 (4)	−0.0075 (3)
S2	0.0299 (4)	0.0516 (4)	0.0691 (5)	−0.0026 (3)	0.0024 (4)	−0.0111 (4)
O1	0.0600 (14)	0.0462 (11)	0.0501 (13)	0.0040 (10)	−0.0039 (10)	−0.0002 (10)
O2	0.0921 (18)	0.0482 (12)	0.0515 (14)	0.0146 (12)	0.0067 (12)	−0.0035 (10)
N1	0.0269 (12)	0.0358 (11)	0.0400 (12)	−0.0034 (9)	−0.0015 (10)	−0.0034 (10)
N2	0.0376 (13)	0.0370 (11)	0.0480 (15)	−0.0003 (10)	−0.0085 (11)	−0.0055 (10)
C1	0.0239 (13)	0.0399 (13)	0.0428 (16)	−0.0024 (11)	−0.0024 (12)	−0.0071 (12)
C2	0.0284 (14)	0.0413 (14)	0.0449 (17)	−0.0015 (11)	0.0032 (13)	−0.0034 (12)
C3	0.0335 (15)	0.0419 (15)	0.064 (2)	−0.0063 (12)	0.0084 (14)	−0.0060 (14)
C4	0.0315 (16)	0.0516 (17)	0.065 (2)	−0.0068 (13)	0.0022 (14)	−0.0210 (15)
C5	0.0442 (19)	0.0621 (19)	0.0462 (18)	−0.0059 (15)	−0.0053 (14)	−0.0130 (15)
C6	0.0360 (16)	0.0484 (15)	0.0424 (17)	−0.0044 (12)	−0.0028 (13)	−0.0027 (13)
C7	0.067 (2)	0.0509 (17)	0.0483 (19)	0.0009 (15)	0.0060 (16)	0.0016 (14)
C8	0.057 (2)	0.067 (2)	0.084 (3)	−0.0187 (17)	0.0041 (19)	−0.0321 (19)
C9	0.075 (2)	0.068 (2)	0.0420 (19)	−0.0127 (18)	−0.0066 (17)	0.0043 (16)
C10	0.0345 (15)	0.0360 (13)	0.0364 (15)	−0.0026 (11)	−0.0045 (12)	0.0027 (11)
C11	0.0473 (18)	0.0419 (15)	0.0472 (18)	0.0015 (13)	−0.0078 (14)	−0.0055 (13)
C12	0.0338 (16)	0.0385 (14)	0.063 (2)	0.0005 (12)	0.0006 (14)	−0.0089 (13)
C13	0.0435 (17)	0.0489 (15)	0.0472 (18)	0.0094 (14)	−0.0111 (14)	−0.0064 (13)
C14	0.0398 (17)	0.0446 (15)	0.0488 (19)	0.0032 (12)	−0.0093 (14)	−0.0020 (14)

### Geometric parameters (Å, °)

**Table:**

S1—C10	1.752 (2)	C6—C9	1.510 (4)
S1—C11	1.841 (3)	C13—C14	1.510 (4)
S2—C10	1.670 (3)	C3—H3	0.9300
O1—C14	1.323 (4)	C5—H5	0.9300
O2—C14	1.198 (4)	C7—H7A	0.9600
O1—H1	0.8200	C7—H7B	0.9600
N1—C1	1.452 (3)	C7—H7C	0.9600
N1—C12	1.502 (3)	C8—H8A	0.9600
N1—C10	1.335 (3)	C8—H8B	0.9600
N2—C12	1.428 (3)	C8—H8C	0.9600
N2—C13	1.454 (3)	C9—H9A	0.9600
N2—C11	1.429 (4)	C9—H9B	0.9600
C1—C6	1.396 (4)	C9—H9C	0.9600
C1—C2	1.387 (4)	C11—H11A	0.9700
C2—C3	1.396 (4)	C11—H11B	0.9700
C2—C7	1.502 (4)	C12—H12A	0.9700
C3—C4	1.390 (5)	C12—H12B	0.9700
C4—C5	1.381 (4)	C13—H13A	0.9700
C4—C8	1.511 (4)	C13—H13B	0.9700
C5—C6	1.387 (4)		
			
S1···C11^i^	3.561 (3)	C14···C3^iv^	3.526 (4)
S1···S1^i^	3.7226 (11)	C14···C2^iv^	3.561 (4)
S2···O1^ii^	3.187 (2)	C1···H1^ii^	3.0800
S2···C6	3.540 (3)	C7···H12B	2.8800
S1···H7A^iii^	3.2000	C10···H9B	2.8000
S1···H3^iv^	3.1100	C12···H7B	2.8300
S1···H13B	2.8500	C14···H11B	2.6900
S2···H12A^v^	2.8300	H1···S2^iv^	2.3700
S2···H9A^vi^	3.0900	H1···C1^iv^	3.0800
S2···H9B	3.0000	H3···H7A	2.3200
S2···H1^ii^	2.3700	H3···H8A	2.3500
O1···C3^iv^	3.352 (3)	H3···S1^ii^	3.1100
O1···S2^iv^	3.187 (2)	H5···H9A	2.3600
O1···C2^iv^	3.367 (3)	H5···O1^ix^	2.9100
O2···N2	2.756 (3)	H7A···H3	2.3200
O2···C11	3.094 (4)	H7A···S1^x^	3.2000
O1···H5^vii^	2.9100	H7B···N1	2.8400
O2···H11B	2.5300	H7B···C12	2.8300
O2···H11A^viii^	2.5500	H7B···H12B	2.1700
O2···H12B^viii^	2.6400	H7B···H7C^x^	2.3800
O2···H7C^iv^	2.8600	H7C···N1	2.8600
N2···O2	2.756 (3)	H7C···H7B^iii^	2.3800
N2···C11^viii^	3.357 (4)	H7C···O2^ii^	2.8600
N1···H9B	2.6900	H8A···H3	2.3500
N1···H7B	2.8400	H8B···H8C^vi^	2.3100
N1···H7C	2.8600	H8C···H8B^xi^	2.3100
N2···H11A^viii^	2.7400	H9A···H5	2.3600
C2···O1^ii^	3.367 (3)	H9A···S2^xi^	3.0900
C2···C14^ii^	3.561 (4)	H9B···S2	3.0000
C3···O1^ii^	3.352 (3)	H9B···N1	2.6900
C3···C14^ii^	3.526 (4)	H9B···C10	2.8000
C6···S2	3.540 (3)	H11A···H12B	2.3400
C7···C12	3.345 (4)	H11A···O2^viii^	2.5500
C7···C10	3.573 (4)	H11A···N2^viii^	2.7400
C9···C10	3.371 (4)	H11B···O2	2.5300
C10···C7	3.573 (4)	H11B···C14	2.6900
C10···C9	3.371 (4)	H12A···S2^xii^	2.8300
C10···C13	3.520 (4)	H12A···H13A	2.2700
C11···S1^i^	3.561 (3)	H12B···C7	2.8800
C11···O2	3.094 (4)	H12B···H7B	2.1700
C11···N2^viii^	3.357 (4)	H12B···H11A	2.3400
C11···C11^viii^	3.577 (4)	H12B···O2^viii^	2.6400
C12···C7	3.345 (4)	H13A···H12A	2.2700
C13···C10	3.520 (4)	H13B···S1	2.8500
			
C10—S1—C11	102.97 (13)	C6—C5—H5	119.00
C14—O1—H1	109.00	C2—C7—H7A	109.00
C1—N1—C12	113.8 (2)	C2—C7—H7B	109.00
C10—N1—C12	125.4 (2)	C2—C7—H7C	109.00
C1—N1—C10	120.8 (2)	H7A—C7—H7B	109.00
C11—N2—C12	111.1 (2)	H7A—C7—H7C	109.00
C12—N2—C13	116.6 (2)	H7B—C7—H7C	109.00
C11—N2—C13	115.3 (2)	C4—C8—H8A	109.00
N1—C1—C2	119.2 (2)	C4—C8—H8B	109.00
C2—C1—C6	122.4 (2)	C4—C8—H8C	109.00
N1—C1—C6	118.3 (2)	H8A—C8—H8B	109.00
C1—C2—C3	117.5 (3)	H8A—C8—H8C	109.00
C1—C2—C7	122.4 (2)	H8B—C8—H8C	109.00
C3—C2—C7	120.1 (2)	C6—C9—H9A	109.00
C2—C3—C4	122.0 (3)	C6—C9—H9B	109.00
C3—C4—C5	118.3 (3)	C6—C9—H9C	109.00
C5—C4—C8	121.1 (3)	H9A—C9—H9B	109.00
C3—C4—C8	120.6 (3)	H9A—C9—H9C	109.00
C4—C5—C6	122.2 (3)	H9B—C9—H9C	109.00
C1—C6—C5	117.7 (3)	S1—C11—H11A	109.00
C1—C6—C9	121.6 (2)	S1—C11—H11B	109.00
C5—C6—C9	120.8 (3)	N2—C11—H11A	109.00
S1—C10—S2	113.48 (16)	N2—C11—H11B	109.00
S1—C10—N1	120.62 (19)	H11A—C11—H11B	108.00
S2—C10—N1	125.84 (19)	N1—C12—H12A	108.00
S1—C11—N2	112.5 (2)	N1—C12—H12B	108.00
N1—C12—N2	115.4 (2)	N2—C12—H12A	108.00
N2—C13—C14	111.2 (2)	N2—C12—H12B	108.00
O1—C14—C13	110.5 (2)	H12A—C12—H12B	107.00
O2—C14—C13	125.3 (3)	N2—C13—H13A	109.00
O1—C14—O2	124.2 (2)	N2—C13—H13B	109.00
C2—C3—H3	119.00	C14—C13—H13A	109.00
C4—C3—H3	119.00	C14—C13—H13B	109.00
C4—C5—H5	119.00	H13A—C13—H13B	108.00
			
C11—S1—C10—S2	176.80 (15)	N1—C1—C2—C3	−176.9 (2)
C11—S1—C10—N1	−5.7 (2)	N1—C1—C2—C7	4.5 (4)
C10—S1—C11—N2	35.4 (2)	C6—C1—C2—C3	−1.6 (4)
C10—N1—C1—C2	−98.6 (3)	C6—C1—C2—C7	179.9 (3)
C10—N1—C1—C6	85.9 (3)	N1—C1—C6—C5	177.9 (2)
C12—N1—C1—C2	77.9 (3)	N1—C1—C6—C9	−2.3 (4)
C12—N1—C1—C6	−97.7 (3)	C2—C1—C6—C5	2.6 (4)
C1—N1—C10—S1	178.62 (18)	C2—C1—C6—C9	−177.7 (3)
C1—N1—C10—S2	−4.3 (3)	C1—C2—C3—C4	−0.5 (4)
C12—N1—C10—S1	2.6 (3)	C7—C2—C3—C4	178.1 (3)
C12—N1—C10—S2	179.7 (2)	C2—C3—C4—C5	1.4 (4)
C1—N1—C12—N2	155.3 (2)	C2—C3—C4—C8	−178.1 (3)
C10—N1—C12—N2	−28.4 (4)	C3—C4—C5—C6	−0.3 (4)
C12—N2—C11—S1	−63.8 (3)	C8—C4—C5—C6	179.2 (3)
C13—N2—C11—S1	71.7 (2)	C4—C5—C6—C1	−1.6 (4)
C11—N2—C12—N1	60.6 (3)	C4—C5—C6—C9	178.7 (3)
C13—N2—C12—N1	−74.3 (3)	N2—C13—C14—O1	−175.4 (2)
C11—N2—C13—C14	72.2 (3)	N2—C13—C14—O2	4.7 (4)
C12—N2—C13—C14	−154.8 (2)		

Symmetry codes: (i) −*x*, −*y*, −*z*; (ii) −*x*+1/2, *y*+1/2, *z*; (iii) *x*−1/2, −*y*+1/2, −*z*; (iv) −*x*+1/2, *y*−1/2, *z*; (v) *x*−1, *y*, *z*; (vi) *x*−1/2, *y*, −*z*+1/2; (vii) −*x*+1, *y*−1/2, −*z*+1/2; (viii) −*x*+1, −*y*, −*z*; (ix) −*x*+1, *y*+1/2, −*z*+1/2; (x) *x*+1/2, −*y*+1/2, −*z*; (xi) *x*+1/2, *y*, −*z*+1/2; (xii) *x*+1, *y*, *z*.

### Hydrogen-bond geometry (Å, °)

**Table:**

*D*—H···*A*	*D*—H	H···*A*	*D*···*A*	*D*—H···*A*
O1—H1···S2^iv^	0.82	2.37	3.187 (2)	174

Symmetry codes: (iv) −*x*+1/2, *y*−1/2, *z*.
